# A Deep Learning Approach to Non-linearity in Wearable Stretch Sensors

**DOI:** 10.3389/frobt.2019.00027

**Published:** 2019-05-08

**Authors:** Ben Oldfrey, Richard Jackson, Peter Smitham, Mark Miodownik

**Affiliations:** ^1^CoMPLEX, University College London, London, United Kingdom; ^2^Institute of Making, University College London, London, United Kingdom; ^3^UCL Institute of Orthopaedics & Musculoskeletal Science, Royal National Orthopaedic Hospital, London, United Kingdom; ^4^Department of Orthopaedics and Trauma, Royal Adelaide Hospital, Adelaide, SA, Australia; ^5^Discipline of Orthopaedics and Trauma, University of Adelaide, Adelaide, SA, Australia; ^6^Department of Mechanical Engineering, University College London, London, United Kingdom

**Keywords:** sensors, real-time, flexible, deep learning, long short term memory neural network

## Abstract

There is a growing need for flexible stretch sensors to monitor real time stress and strain in wearable technology. However, developing stretch sensors with linear responses is difficult due to viscoelastic and strain rate dependent effects. Instead of trying to engineer the perfect linear sensor we take a deep learning approach which can cope with non-linearity and yet still deliver reliable results. We present a general method for calibrating highly hysteretic resistive stretch sensors. We show results for textile and elastomeric stretch sensors however we believe the method is directly applicable to any physical choice of sensor material and fabrication, and easily adaptable to other sensing methods, such as those based on capacitance. Our algorithm does not require any a priori knowledge of the physical attributes or geometry of the sensor to be calibrated, which is a key advantage as stretchable sensors are generally applicable to highly complex geometries with integrated electronics requiring bespoke manufacture. The method involves three-stages. The first stage requires a calibration step in which the strain of the sensor material is measured using a webcam while the electrical response is measured via a set of arduino-based electronics. During this data collection stage, the strain is applied manually by pulling the sensor over a range of strains and strain rates corresponding to the realistic in-use strain and strain rates. The correlated data between electrical resistance and measured strain and strain rate are stored. In the second stage the data is passed to a Long Short Term Memory Neural Network (LSTM) which is trained using part of the data set. The ability of the LSTM to predict the strain state given a stream of unseen electrical resistance data is then assessed and the maximum errors established. In the third stage the sensor is removed from the webcam calibration set-up and embedded in the wearable application where the live stream of electrical resistance is the only measure of strain-this corresponds to the proposed use case. Highly accurate stretch topology mapping is achieved for the three commercially available flexible sensor materials tested.

## Introduction

Measuring real time stress and strain in wearable technology is a key requirement because this information is required to monitor the recovery of a shoulder operation through the wearing of a therapeutic garment, or the stretch of a hamstring of an athlete during training, or to protect the vulnerable skin of those who wear prosthetics or orthotics (de la Fuente et al., 2000; Howe and Sherwood, 2009). On the face of it measuring stretch should be relatively easy, especially because basic stretch sensors have been around for a long time. However, the non-linearity and strain rate dependent hysteresis of high strain flexible sensors have proved difficult issues to solve (Amjadi et al., 2016; Noh, 2016; Seshadri et al., 2016).

In this paper we recognize that developing stretch sensors with linear responses is difficult and that viscoelastic effects and strain rate effects are often unavoidable. Instead of trying to engineer the perfect linear sensor we take a different approach. We present a deep learning method that can learn the peculiarities of the non-linearity of cheap and easy-to-make sensors, while still giving reliable and robust strain data. This way we can offset the disadvantages of some types of sensors, while maintaining their advantageous simplicity in other areas. The method is entirely general and we believe it can be used with any flexible stretch sensor.

**Figure 1:**
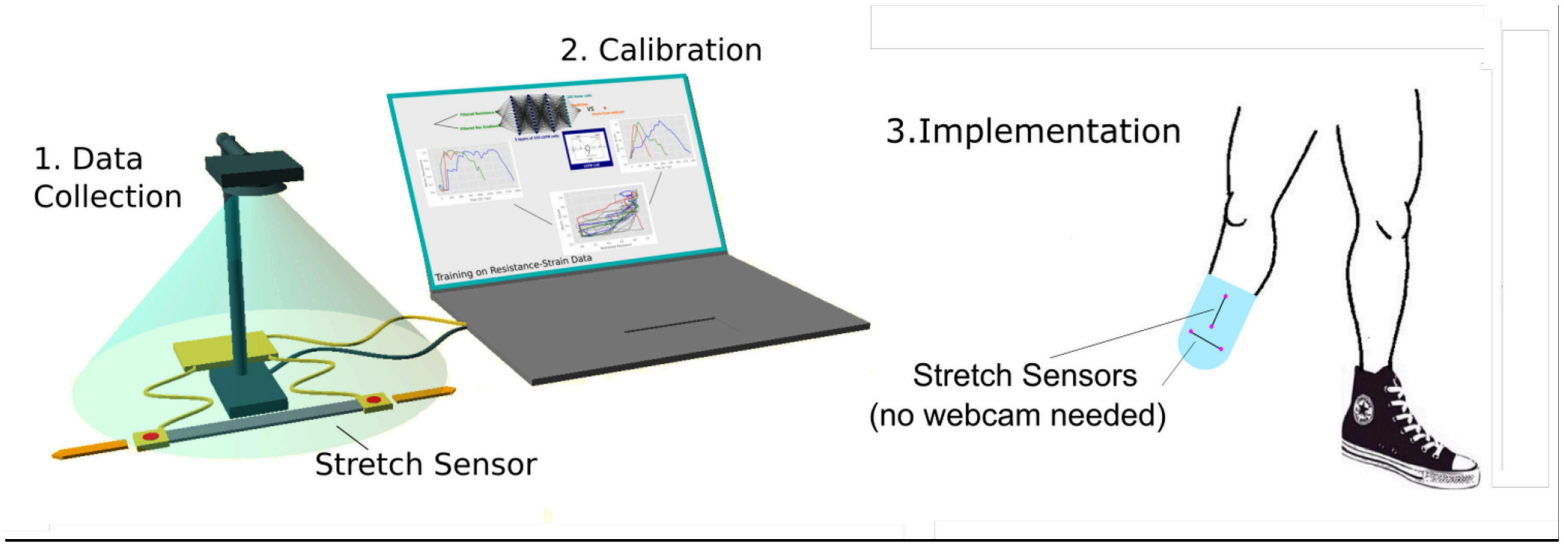
Overview of System showing our three-stage approach to developing a wearable sensor. The first stage involves a calibration step in which the sensor material is measured using a webcam while the electrical response is measured via a set of arduino-based electronics. In the second stage the data is passed to a Long Short Term Memory Neural Network which is trained using part of the data set. In the third stage the sensor is removed from the webcam calibration set-up and embedded in the wearable technology where the live stream of electrical resistance is the only measurement taken - this corresponds to the proposed use case.

We take a three-stage approach to developing a wearable sensor. The first stage involves a calibration step in where the strain of a sensor is measured using a webcam while the electrical response is measured via a set of arduino-based electronics. This data collection stage is designed to heed clinical advice that strains applied manually, over a range of strains and strain rates, mimics the real use cases of wearables which will always involve highly varying strain rates. This is the reason we did not use mechanically driven stretching methods for data collection. The correlated data between electrical resistance, measured strain and strain rate are stored, see Figure 1.

In the second stage, the data is passed to a Long Short Term Memory Neural Network (LSTM) which is trained using part of the data set. The ability of the LSTM to predict the strain state given a stream of unseen electrical resistance data is then assessed, see Figure 1.

In the third stage, the sensor is removed from the webcam calibration set-up and embedded in the wearable technology where the live stream of electrical resistance is the only measure of strain-this corresponds to the proposed use case. We are currently developing the approach to deal with stress and pressure sensors, as well as 2D shear sensors but as proof of principle we have focused on 1D stretch sensors in this paper. Nevertheless, there are numerous applications where the measurement of linear 1D stretch is desired, but not currently available to practitioners. For example in the fitting of orthotic and prosthetic liners where the information on the expansion and expansion rate greatly affect comfort and skin health, as shown in Figure 1. Here, In these cases multiple 1D sensors can combine to give important information. More commonly, simple body tracking is required, such as the tracking of position of the arm, this could be done with a linear stretch sensor placed on the elbow, as if the material used is sufficiently thin, then change in resistance due to bending is negligible.

We used this procedure to investigate three different commercially available flexible sensor materials, Medtex P130+B and Techniktex P130+B both from Statex Produktions & Vertriebs GmbH, and Adafruit Conductive Rubber Adafruit Industries. We show that in each case our deep learning approach provides robust strain information with smaller errors than other methods.

## Background

There is a large body of research carried out to produce new flexible sensors, these fall broadly into two categories, resistive and capacitive sensors.

### Capacitive Strain Sensing

Capacitive stretch sensors are typically fabricated by sandwiching a dielectric between two electrode layers, all of which need to be stretchable. The impedance is measured by analyzing the response variable signal frequency, and from this the capacitance is estimated without needing to have any information about the resistance change in the conductive electrode layers. The advantage of capacitive flexible sensors is their linearity and high sensitivity. However, they tend to have low gauge factors and because of their layered structure are more complex to fabricate. Nevertheless, capacitive sensors have been successfully made using conductive silicone for measuring pressure and shear stresses simultaneously at the stump-socket interface of lower-limb amputees (Laszczak et al., 2015, 2016). Conductive fabrics have been used by Andreas Tairych (2017) to create multiple capacitive stretch sensors requiring only one channel for measurement; Atalay et al. (2017) used conductive stretch fabric as the electrodes sandwiching a silicone dielectric for a customizable strain sensor for human motion tracking; Kappel et al. (2012) developed a strain sensor based on a dielectric electro-active polymer (DEAP) that acts as an elastic capacitive material, strainable in one direction for measuring in-shoe navicular drop during gait; Zens et al. (2015) used a complex layering of non-conductive PDMS and conductive PDMS made using carbon black particles as a novel approach to dynamic knee laxity measurement (Fassler and Majidi, 2013; Zens et al., 2015) produced soft-matter capacitors and inductors from microchannels of liquid-phase gallium–indium-tin alloy (galinstan) embedded in Ecoflex^Ⓡ^ 00-30.

### Resistive Strain Sensing

The advantage of resistive flexible sensors are their relative simplicity and stability for large strain in excess of 100%, however they tend to be highly non-linear and hysteretic, the two most common complex behaviors being relaxation time and the resistance spikes associated with fast changes in strain rate (Tiwana et al., 2012). There are two major mechanisms by which piezoresistive behavior useful for stretch sensing is achieved. These are: (1) by doping an elastomer matrix with a conductive filler of some kind—this is primarily a nanoscale effect; and (2) by constructing a conductive pathway which undergoes a significant geometrical change under stretch that the resistance also changes—this is primarily a macroscale effect.

For the first type, a polymer with low Young's modulus, such as PDMS, rubber or silicone is used as a matrix and a conductive filler such as metal nanoparticles or carbon allotropes. When the ratio of filler to matrix content is above the percolation threshold, the composite material will conduct electricity. When the material is stretched, this increases the gaps of insulating matrix between adjacent conductive particles reducing the number of possible electron tunneling pathways, thus increasing the electrical resistance. There many ways to fabricate such as materials, for example Boland et al. (2014) describe a simple method to infuse liquid-exfoliated graphene into natural rubber to create conducting composites, displaying 10^4^-fold increases in resistance and working at strains exceeding 800%; Ferreira et al. (2017) report a carbon nanotube (CNT) and PVDF composite capable of measuring the interface pressure within prosthetic stump/sockets; Watthanawisuth et al. (2015) report a novel sensor using a 3D-Graphene foam amalgam with PDMS; Lee et al. (2015) report a sensor from highly stretchable conductive fiber composed of silver nanowires (AgNWs) and silver nanoparticles (AgNPs) embedded in a styrene-butadiene-styrene (SBS) elastomeric matrix capable of 900% strain; Larimi et al achieved 350% strain with a low cost sensor fabricated by infusing graphene nano-flakes into a rubber-like adhesive pad (Larimia et al., 2018). This is similar to the commercially available Adafruit Conductive Rubber sensor Adafruit Industries we tested in this work.

For the second type of piezoresistive sensor, a geometrical change is achieved in a conductive material. This can be a simple change as in a highly conductive liquid in a fluidic channel whose length increases and cross-sectional area decreases, or it can be a much more complex change, such as the change in the conductive pathways of a stretchable fabric. In the latter case, as the textile is stretched and relaxed, different parts of the weave come into contact with each other, making discrete pathway changes on the scale of the weft and weave. There many ways to fabricate such as materials, for example Chossat et al. (2015) describe a complex microchannel network with a room temperature ionic liquid (RTIL); Mengüç et al. (2014) made Ga-In based fluidic strain sensors, but refined their design with the use of discretized stiffness gradients to improve mechanical durability; Michaud et al. (2015). combine thin gold films on silicone which display large reversible change in electrical resistance upon stretching, with eutectic liquid metal conductors to maintain bulk metal conductivity, even upon extensive elongation; Smart fabric sensors (Castano and Flatau, 2014); and smart textiles (Nejad et al., 2017) similar to the commercially available Medtex P130+B and Techniktex P130+B Statex Produktions & Vertriebs GmbH we tested in this work.

## Methods

### Dynamic Electrical Resistance Measurement of the Stretch Sensors

**Figure 2:**
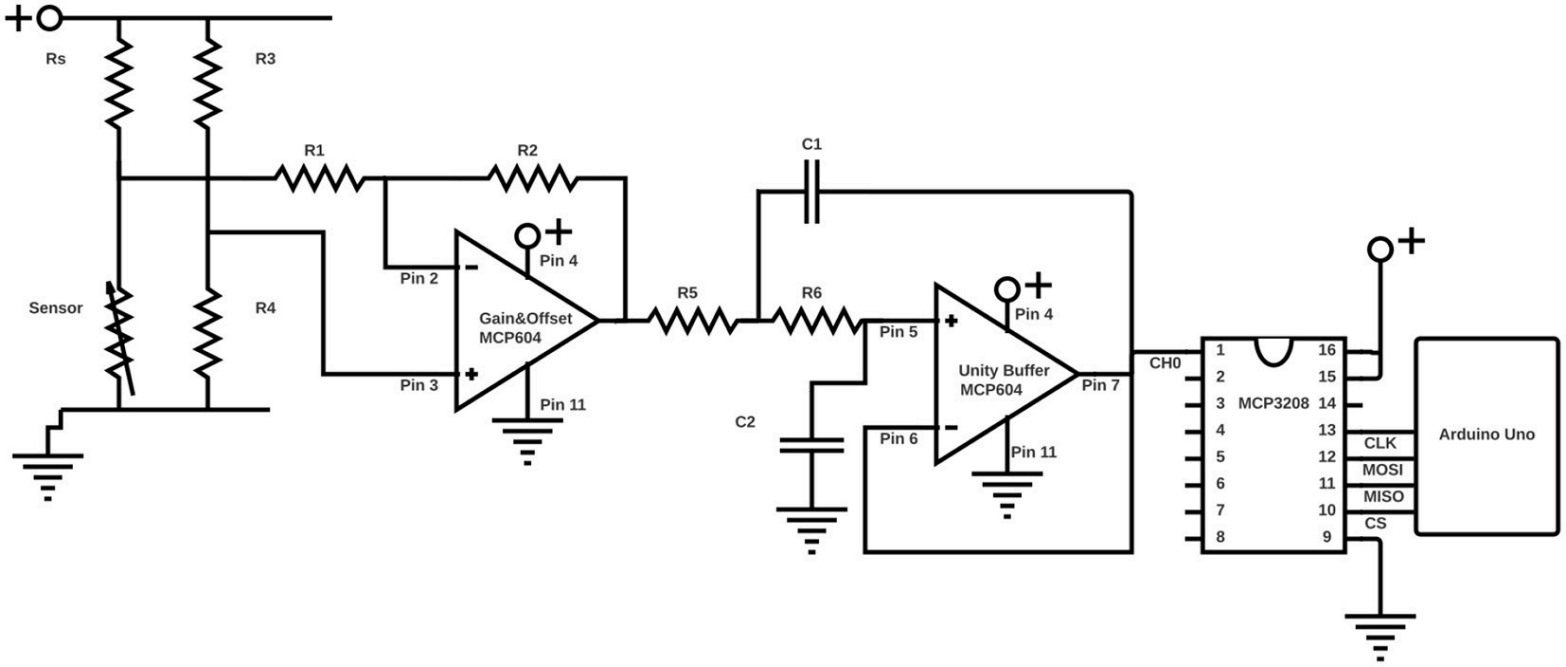
A diagram of the analog signal processing circuit used in this work.

The electrical resistance of each strain sensor was measured using an analog signal processing (ASP) circuit which consists of a voltage divider, operational amplifier, filtering and an ADC as shown in Figure 2. After digitalisation, the signal undergoes digital signal processing (DSP) which consists of an oversampling routine onboard the Arduino, serial communication via USB to laptop, where it goes through a 5th Order Butterworth filter. This creates a data stream of filtered resistance and its gradient. These values are the inputs to the neural network described in the LSTM section. The values of the resistors and capacitors used in the ASP were calculated to optimize the dynamic range, and reduce noise. These calculations are explained in the following sections.

#### ASP: Optimizing Dynamic Range

The opposing resistor, *R*_*s*_ from Figure 2, for the voltage divider is chosen using the maximum and minimum resistance measured by a standard multimeter (these are not necessarily at the max and min stretch). *R*_*s*_ was then calculated using Equation 1:

(1) $$\begin{array}{r} R_{s}=\sqrt{R_{max}\times R_{min}} \end{array}$$

#### ASP: Gain and Offset

The analog conditioning circuit is an inverting op amp configuration using an MCP3208 Chip. The inversion is irrelevant to the analysis of the signal in this application, but has advantages compared to the non-inverting configuration. In particular, the noise contribution is amplified equally with the signal, which is not true for the non-inverting case, in which it is amplified to a greater degree (Carter, 2002). *R*_1_, *R*_2_, *R*_3_, and *R*_4_ from Figure 2 were calculated using Equations 2 and 3. Initial values for *R*_2_ and *R*_3_ must be of the same order of magnitude, and are chosen so to minimize the input resistance to the ADC, which must be kept below 1, 000 Ω for the MCP3208. So that

(2) $$\begin{array}{r} R_{1}=\frac{R_{2}}{\left|m\right|} \end{array}$$

(3) $$\begin{array}{r} R_{4}=\frac{bR_{3}R_{1}}{5\left(R_{2}+R_{1}\right)-bR_{1}} \end{array}$$

where $m=\frac{-5}{V_{rag}}$, *b* = *mV*_*low*_. *V*_*rag*_ is the voltage range of the stretch sensor and optimized voltage divider measured through the ADC, and *V*_*low*_ is the lowest value in this range, with the assumption that *m* is negative, and *b* is positive. A more detailed description of this method can be found in Carter (2002).

#### ASP: Filtering

Frequencies higher than the sampling rate appear as lower frequencies when sampled, which can result in a variety of possible distortions to a voltage signal. To avoid such aliasing, frequencies contained in the signal must be below the Nyquist frequency, which is defined as half the sampling rate. This was achieved by implementing a second order unity gain Sallen-key low-pass filter, for which Equation 4 dictated the choice of resistors and capacitors to achieve the required cut-off frequency.

(4) $$\begin{array}{r} f_{cut-off}=\frac{1}{2\pi \sqrt{R_{5}R_{6}C_{1}C_{2}}} \end{array}$$

For the unity gain op amp used this was simplified as *R*_5_ = *R*_6_ = 470 Ω, and *C*_1_ = *C*_2_ = 0.01 μ*F*.

#### DSP: Oversampling

Oversampling was undertaken onboard the Arduino, so as not to take CPU power away from the laptop, slowing the intensive neural network processing. A useful property of the inverse relationship between sample rate and resolution, is that it holds true even above the physical resolution of the ADC, however the sample rate is reduced by averaging over multiple real samples, here we averaged over *N*_*s*_ samples, to maintain the required sample rate. This achieves a *n* bit increase with $N_{s}=2^{2n}$. A reduction in noise power also resulted from this by a factor of $\frac{1}{N_{s}}$. In this work we use *N*_*s*_ = 25.

The frame rate of the camera is orders of magnitude lower than the sample rate of the ASP, so it governs the size of the data set we could produce, namely 30 frames per second. All digital processes had to be achieved within the $\frac{1}{30}$ of a second frame window, and for this reason the oversampling was performed on board the Arduino, as this could be performed in parallel to computations on the computer CPU. The resistance was filtered using the SciPy package, Version 0.19.1 was used for this study. The filters used were the signal.butter() and signal.filtfilt() functions. The gradient was measured using the gradient() function in the NumPy package, Version 1.15.0 was used for this study.

### Measuring Strain in Real-Time

**Figure 3:**
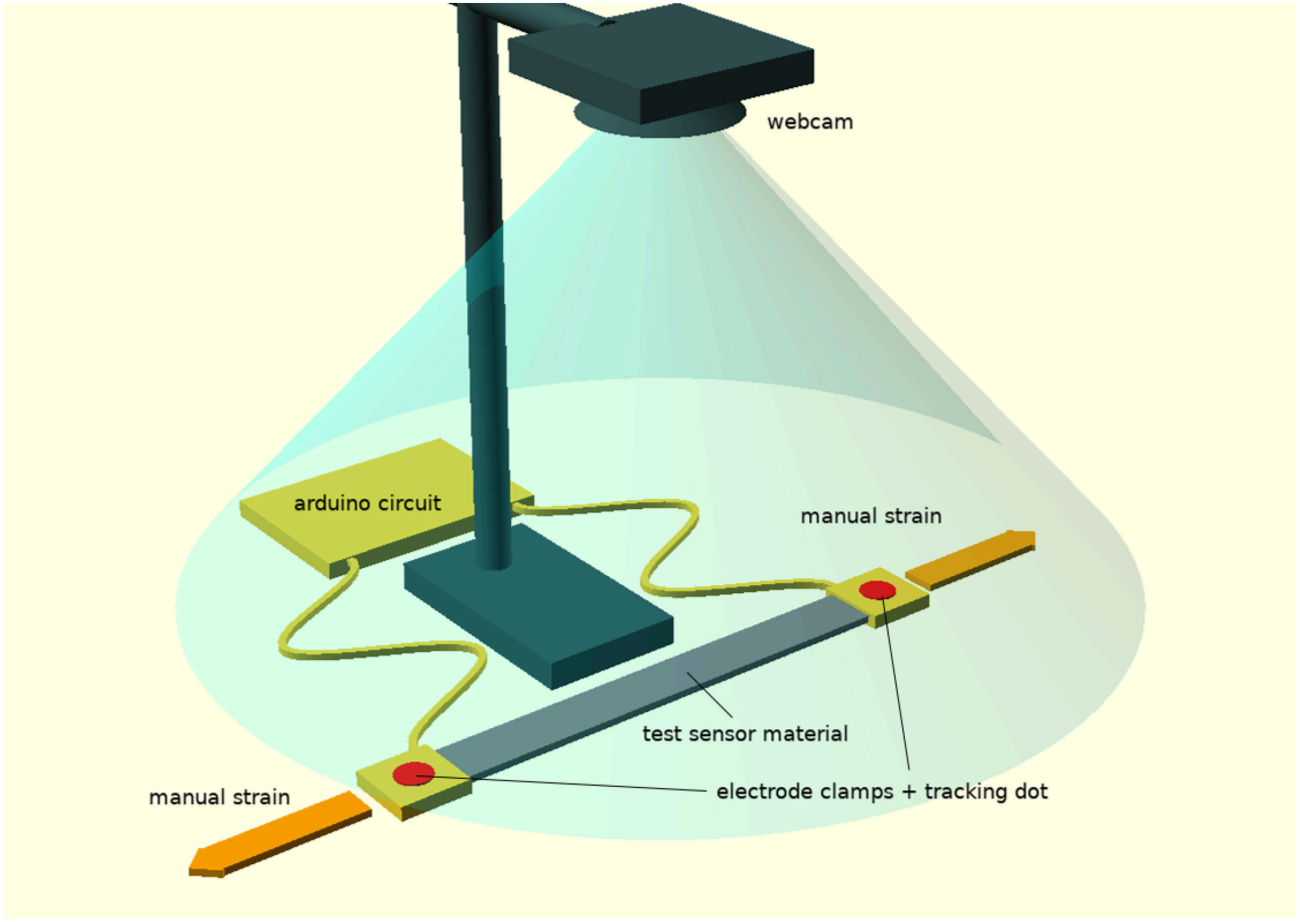
A diagram showing the experimental set-up for the calibration step in our process, which involves the use of a webcam to track dots in real-time on a flexible sensor to measure elongation, while arduino-based electronics is used to collect the correlated electrical data.

To measure real-time strain and resistance, each stretch sensor was positioned under a webcam connected to a laptop, see Figure 3. We used a standard Logitech C270 camera with a frame rate of 30 frames per second. For each experiment the webcam was positioned 50 cm above the flexible sensor using a clamp stand. The flexible sensor was connected electrically to the ASP circuit and red dot labels were placed on the electrode clamps of the flexible sensor. The flexible sensor was then stretched and unstretched manually at a range of strain rates (from 0 to 1 *s*^−1^). The computer vision package OpenCV (Version 3.3.0 was used for this study) running on the laptop was used to collect the images from the camera and automatically detect the red dots and their coordinates, which were used to compute the real-time strain. The strain was correlated with the resistance measurements to produce a data set as input for the LSTM.

### Long Short Term Memory Neural Network (LSTM)

Long Short Term Memory networks are a special kind of Recurrent Neural Network (RNN) introduced by Hochreiter and Urgen Schmidhuber (1997), which are capable of learning long-term dependencies, and have advantages over traditional RNNs, such as avoiding the vanishing gradient problem. Traditional RNNs map input sequences to outputs using the following recurrence equations:

(5) $$\begin{array}{r} h_{t}=g\left(W_{xh}x_{t}+U_{hh}h_{t-1}+b_{h}\right) \end{array}$$

(6) $$\begin{array}{r} z_{t}=g\left(W_{hz}h_{t}+b_{z}\right) \end{array}$$

where *g* is an element-wise non-linearity, e.g., sigmoid function or hyperbolic tangent, *x*_*t*_ is the input matrix at time *t*. *h*_*t*_ is the hidden state matrix which from Equation 5 is a function of the input at the same time step (*x*_*t*_), modified by a specific weight matrix *W*_*xh*_, and the previous hidden state *h*_*t*−1_ modified by its own weight matrix *U*_*hh*_. In Equation 6 the output matrix *z*_*t*_ is determined by a similar process to give an output prediction.

**Figure 4:**
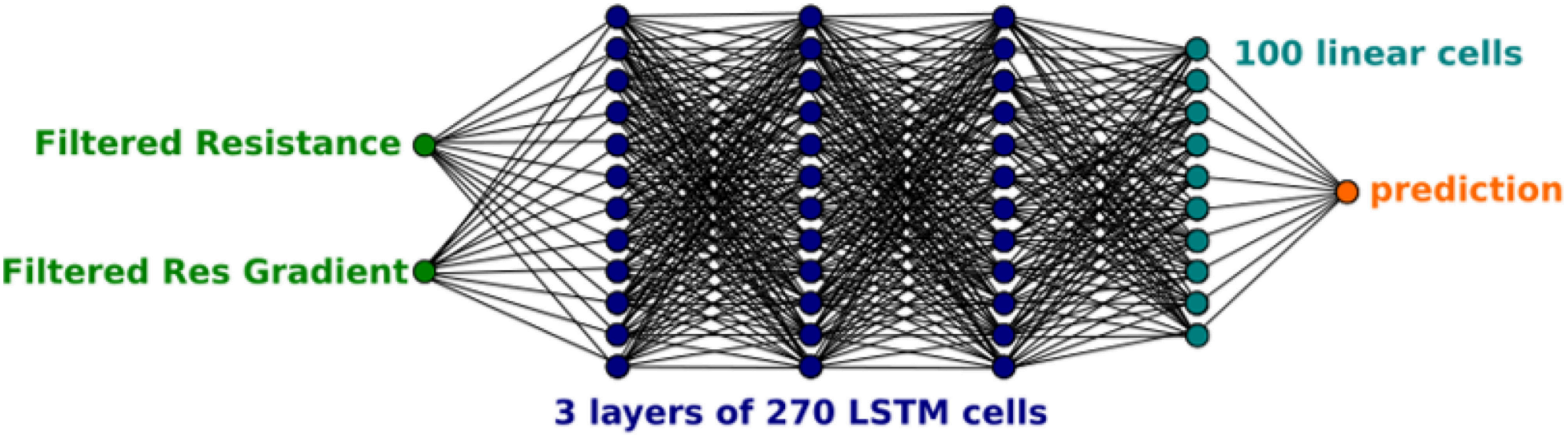
A diagram of the architecture of the neural network used.

**Figure 5:**
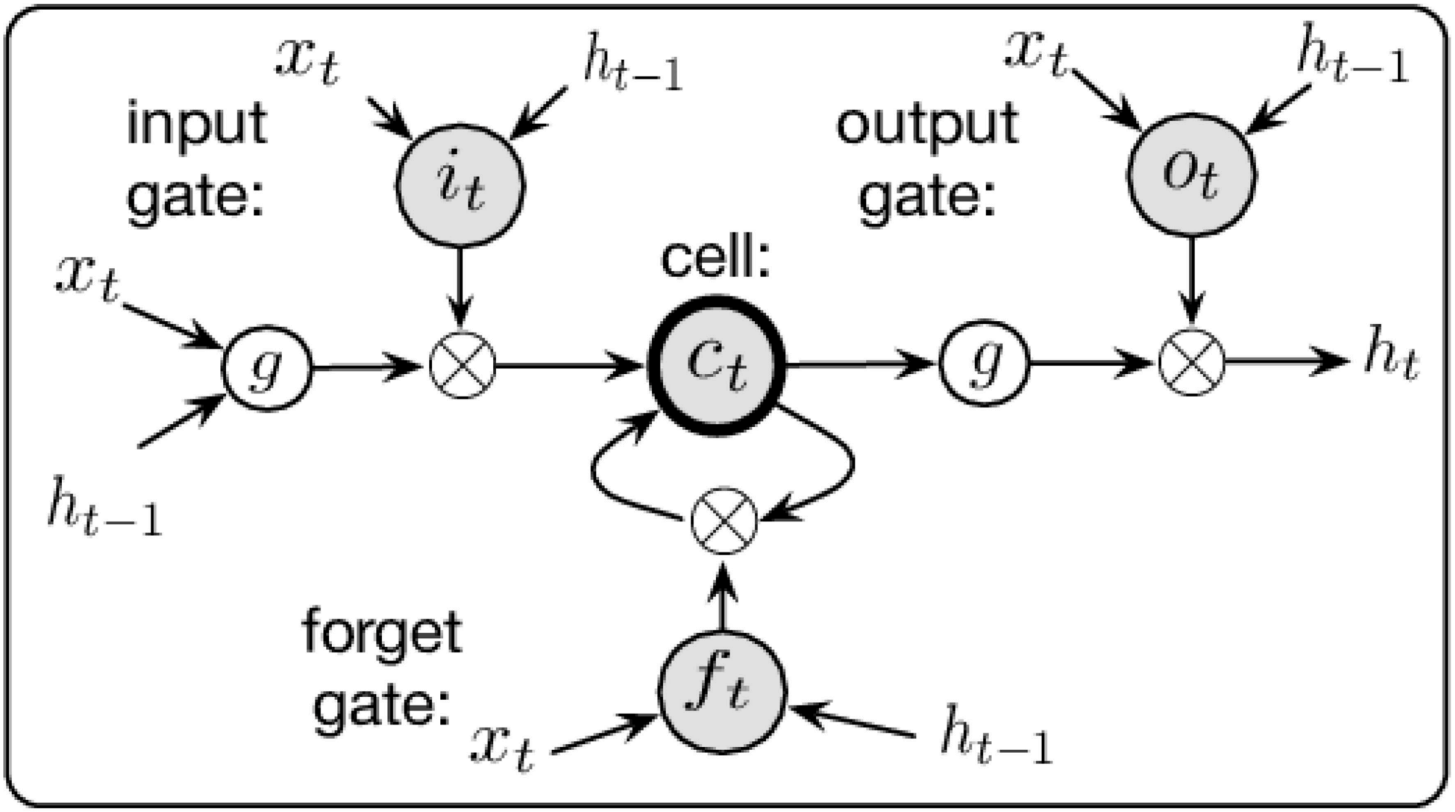
A pictoral representation of the LSTM cell used (Chen and Wang, 2017).

The weighted matrices act as filters to determine the importance of various inputs, and their elements along with the biases *b*_*h*_ and *b*_*z*_ are the parameters in the “deep learning.” The “deep” indicates that there are multiple separate layers with additional hidden states like *h*_*t*_ whose role is to modify the output layer, see Figure 4. LSTMs build on this RNN framework by including memory cells comprising of three types of gates: (i) a Forget Gate, which conditionally decides what information to throw away; (ii) an Input Gate which conditionally decides which values from the input to update to the memory state; and (iii) an Output Gate, which conditionally decides what to output based on input and the memory state, see Figure 5. Each cell is like a mini-state machine where the gates of the cells have weights that are learned during the training procedure. These cells are described mathematically by the following equations:

(7) $$\begin{array}{r} i_{t}=\sigma \left(W_{ix}x_{t}+U_{ih}h_{t-1}+b_{i}\right) \end{array}$$

(8) $$\begin{array}{r} f_{t}=\sigma \left(W_{fx}x_{t}+U_{fh}h_{t-1}+b_{f}\right) \end{array}$$

(9) $$\begin{array}{r} o_{t}=\sigma \left(W_{ox}x_{t}+U_{oh}h_{t-1}+b_{o}\right) \end{array}$$

(10) $$\begin{array}{r} c_{t}=f_{t}⊙c_{t-1}+i_{t}⊙tanh\left(W_{cx}xt+U_{ch}ht-1+b_{c}\right) \end{array}$$

(11) $$\begin{array}{r} h_{t}=o_{t}⊙tanh\left(c_{t}\right) \end{array}$$

where ⊙ is the Hadamard product and *i*_*t*_ is the input gate (state at time *t*), *f*_*t*_ is the forget gate, *o*_*t*_ is the output gate, *c*_*t*_ is the cell state, and *h*_*t*_ is the hidden state analogous to that from the simpler RNN example. A pictoral representation of this LSTM unit can be seen in Figure 5, where as in the simple RNN, the cell is given the input matrix *x*_*t*_ and the previous time step's hidden state *h*_*t*−1_. Here however, they are passed through the three gates, each with separate modifying weight matrices, serving their separate functions, before outputing the hidden state *h*_*t*_. A more detailed description of LSTM architecture can be found in Jozefowicz et al. (2015).

Our network architecture comprises 3 layers of 270 LSTM cells followed by a single linear layer which maps the final recurrent layer to a single output via further weighted & biased matrix multiplication. The architecture of this structure is shown in Figure 4 showing the input nodes, the layers of LSTM cells (each one as shown in Figure 5), the output matrix, and the output node. The network size was set by comparing varied architectures that were able to complete within 1/30 of a second (along with the other processing requirements). This means that once the vision tracking is removed from the system, the sampling rate can be set to match the 30 fps of the original data collection, preserving the accuracy of the predictions without any time lag.

The weights and biases of the 4 network layers are trained by gradient descent using Adaptive Moment Estimation that computes adaptive learning rates for each parameter (Kingma and Ba, 2014). The loss function used in this method is assessed by calculating the root mean squared error (RMSE) of the set of batch predictions vs. the actual strain measurements tracked with the webcam.

#### Dropout, Noise, Processing and Implementation

Dropout is a recently introduced regularization method as described by Srivastava et al. (2014), which has been very successively applied to standard feed-forward neural networks, but with less success when applied to recurrent networks. Dropout entails probabilistically excluding a given proportion of the input and internal connections from activation and weight updates while training the network. Our approach follows the method suggested by Zaremba et al. (2014) for applying it to LSTM networks, which in short, only applies it to all non-recurrent connections in the cell structure.

LSTM networks produce better results on larger datasets and many training steps. In order to train them effectively using small datasets, we create more data for it to use, by adding symmetric noise to the inputs and continuing training. With this larger augmented dataset, a much greater number of useful training steps are possible. It also serves as an effective regularization method, reducing overfitting of the network, which is a primary concern for all networks, particularly those with smaller datasets.

A Butterworth filter is used with a cut-off frequency dictated by the highest measured frequency present in the vision data. This enables usable predictions to be achieved even when the neural network is not able to reduce its loss function to a low enough value to produce stable outputs. In general, the raw network output is noisy, however after filtering, shows excellent calibration correlation.

This method was implemented using the Tensorflow package, Version 1.8 was used for this study, on a Toshiba Tecra laptop running an Arch based linux operating system.

### Materials

We tested three commercially available stretch sensor materials with our method, these were Medtex P130+B and Techniktex P130+B both from Statex Produktions & Vertriebs GmbH, and Adafruit Conductive Rubber Adafruit Industries.

#### Medtex P130+B

Medtex P130+B is a commercially available stretch nylon, interweaved with silver, aimed at medical dressings due to the anti-bacterial properties of silver. It is also used to construct stretchable conductive circuits and basic stretch sensors. Compared to solid rubber-like materials, it has a much smaller relaxation time than some material options, yet does display some additional complex resistive behavior which makes more precise use more difficult.

A 15cm sized strip of the Medtex P130+B fabric was cut and placed in metal clamps attached to the ASP circuit. The maximum and minimum resistance values were measured as 41 and 153 Ω. Using Equation 1, the optimum resistance value was found to be 79.2 Ω, therefore a 75 Ω resistor was used for *R*_*s*_, as this was the closest single standard value available. The sensor was then stretched to find the operating range of this set-up, which was found to be 1.52−2.79 *V*, a span of 1.27 *V*. The required gain was calculated to be −3.94 *V*/*V*, with an off-set of 5.99 *V*. Using Equations 2 and 3 the resistor values were calculated to optimize the ASP circuit for this experiment and found to be: *R*_1_ = 130 Ω, *R*_2_ = 510 Ω, *R*_3_ = 510 Ω, *R*_4_ = 160 Ω.

#### Techniktex P130+B

Techniktex P130+B is an advanced conductive fabric aimed at the wearable electronics market. It claims to have homogeneous conductivity in all directions, and have more reliable and linear behavior. It still has an associated relaxation time, however it only increases resistance as it is stretched, unlike the Medtex fabric.

A 15 cm sized strip of the Techniktex fabric sample was cut and placed in metal clamps attached to the ASP circuit. The maximum and minimum resistance values were measured as 16 and 28 Ω. Using Equation 1, the optimum resistance value was found to be 21.2 Ω, therefore a 22 Ω resistor was used, as this was the closest single standard value available. The sensor was then stretched to find the operating range of this set-up, which was 1.95−2.45 *V*, a span of 0.5 *V*. The required gain was calculated to be −10 *V*/*V*, with an off-set of 11 *V*. Using Equations 2 and 3 the resistor values were calculated to optimize the ASP circuit for this experiment and found to be: *R*_1_ = 51 Ω, *R*_2_ = 510 Ω, *R*_3_ = 510 Ω, *R*_4_ = 120 Ω.

#### Adafruit Conductive Rubber

The Adafruit conductive rubber comes in an 3 mm diameter extruded cord, a 15 cm sized length was placed in metal clamps attached to the ASP circuit. The maximum and minimum resistance values were measured as 1.18 and 2.6 *k*Ω. Using Equation 1, the optimum resistance value was found to be 1.75 *k*Ω, therefore a 22 *k*Ω resistor was used, as this was the closest single standard value available. The sensor was then stretched to find the operating range of this set-up, which was 2−2.6 *V*, a span of 0.6 *V*. The required gain was calculated to be −8.3 *V*/*V*, with an off-set of 16.7. Using Equations 2 and 3 the resistor values were calculated to optimize the ASP circuit for this experiment and found to be: *R*_1_ = 62 Ω, *R*_2_ = 510 Ω, *R*_3_ = 510 Ω, *R*_4_ = 330 Ω.

## Results

For each of the three stretch sensors the same experimental method was carried out. This comprised of collecting data by manually pulling and releasing the sensors in a manner consistent with real usage i.e., over a period of many minutes and with a range of strain rates. For each material some of the correlated sets of measured strain from the webcam and measured electrical resistance were used to train the neural network. Subsequently the neural network was used to predict the strain when only supplied with unseen electrical resistance data which was then compared with the unseen measured strain and the errors computed. The results are as follows.

### Medtex P130+B

**Figure 6:**
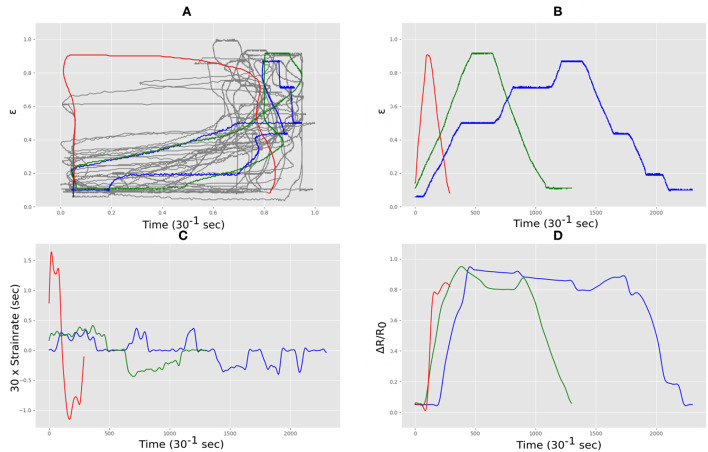
Strain and electrical resistance behavior of the Medtex P130+B during stretching; **(A)** normalized strain vs. normalized resistance; **(B)** normalized strain vs. time; **(C)** strain rate vs. time; **(D)** normalized resistance vs. time.

The sensor was manually stretched and relaxed for ~15 min under the webcam, producing ~27,500 correlations of measured sensor length vs. measured electrical resistance. The data was pre-processed to produce a set of filtered resistance and unfiltered resistance gradients, matched against values of strain. The complex nature of this data can be seen in Figure 6A where three stretch and relax cycles for the sensor with three different strain rates (depicted in red, blue and green) produced very different functional forms (the gray line shows all the data). Figure 6B shows the measured strain correlated with the measured electrical resistance showing that the strain rates were different in each case and varied in a realistic i.e., non-linear manner. The strain rates are quantified in Figure 6C which shows the characteristic spikes which are typical of such flexible stretch sensors. The rate of change of resistance for each case is shown in Figure 6D which was used as an input for the LSTM neural network.

**Figure 7:**
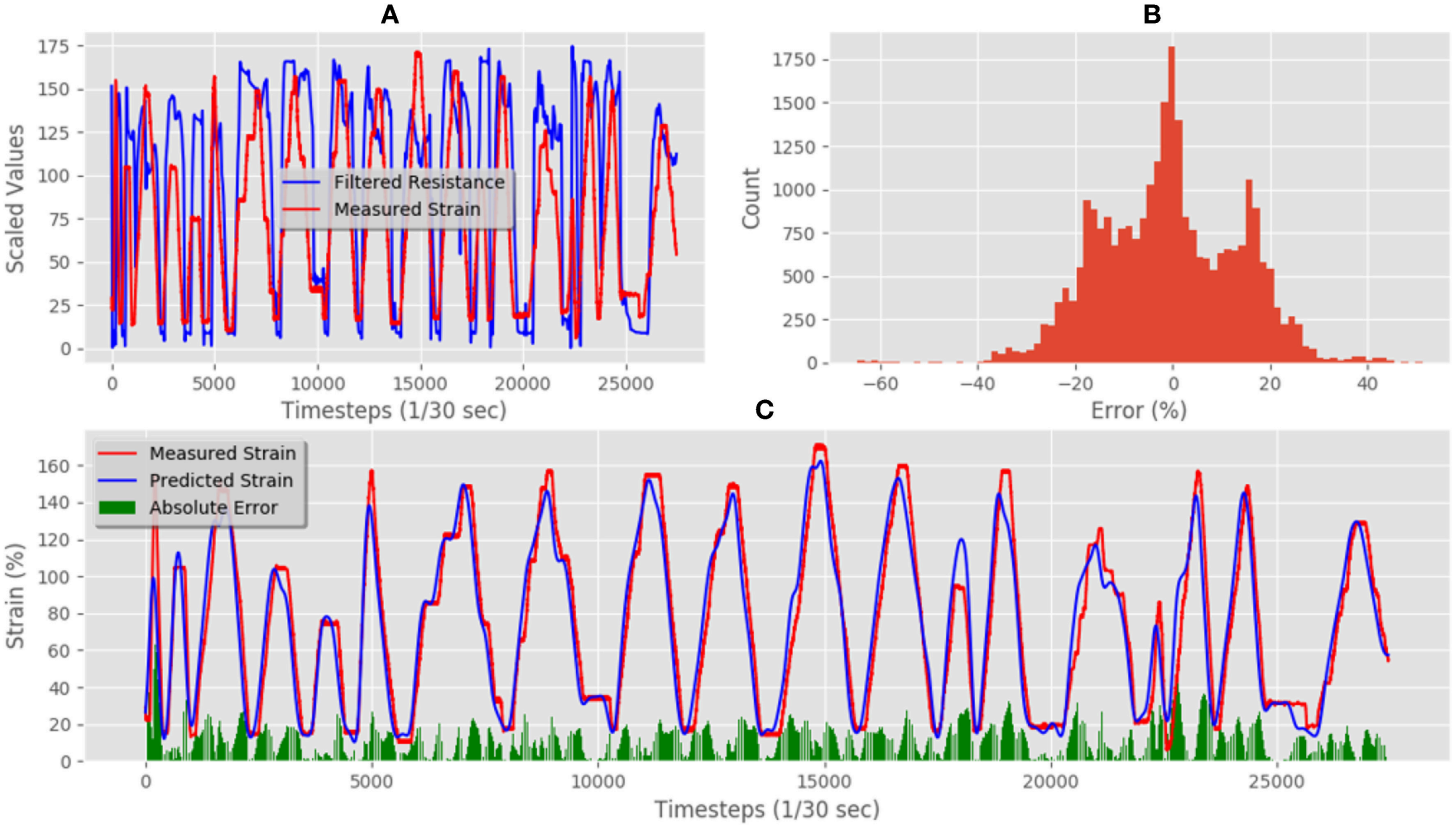
Medtex P130+B training data predictions and errors: **(A)** electrical resistance and correlated strain of training set vs. time; **(B)** training set error distribution; **(C)** Measured strain vs. predicted strain for training set.

This dataset was used to train our LSTM architecture for ~17 h following an automated training schedule. The correlation between measured strain and resistance of the training data set can be seen in Figure 7A. Figure 7B shows the correlation between measured and predicted strain in the final trained network. The Mean Absolute Error (MAE) between prediction and measured was 11.38% total strain, and the error distribution can be seen in Figure 7C. The correlation was calculated using the Pearson Product-Moment Correlation.

**Figure 8:**
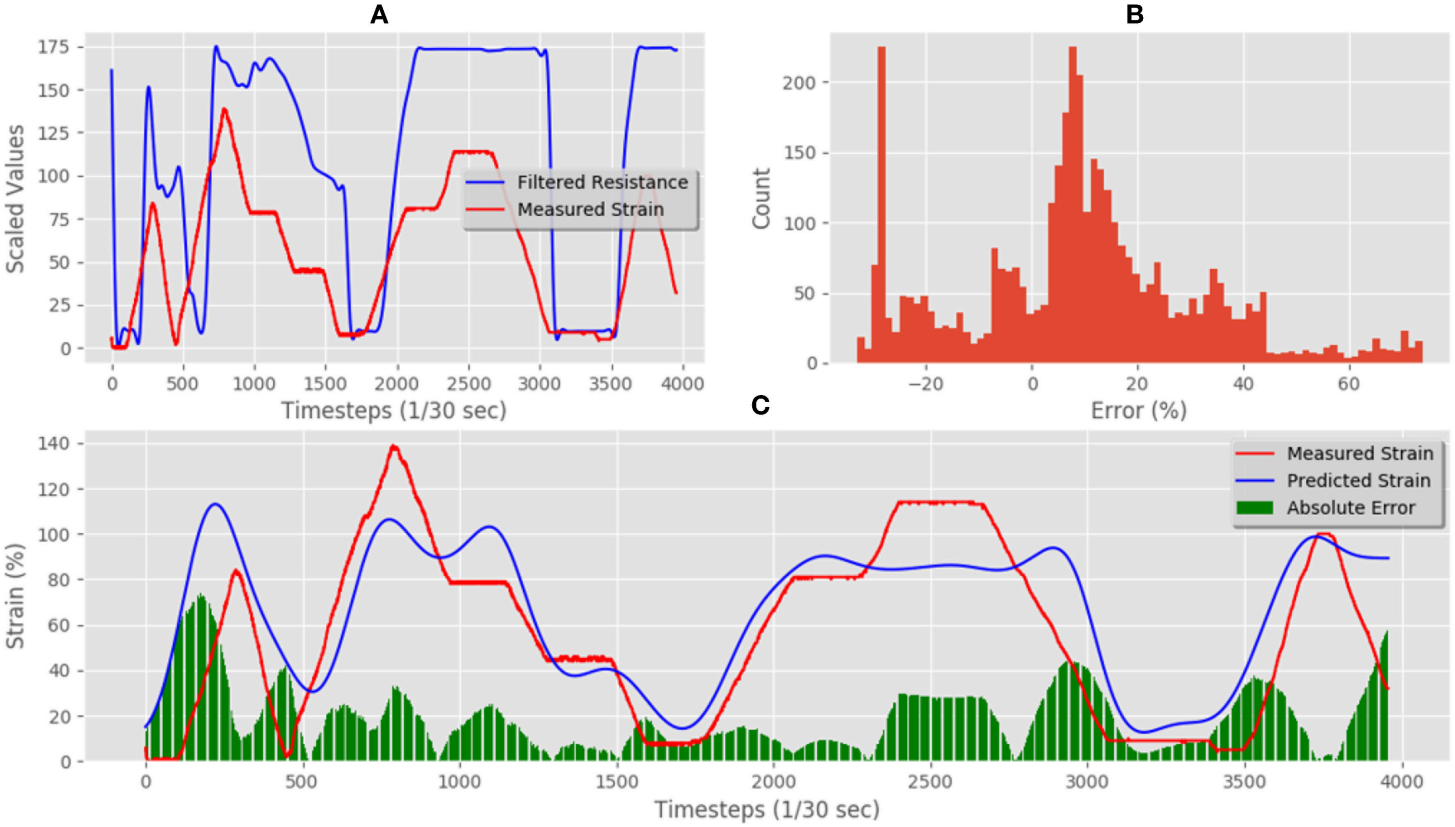
Medtex P130+B real-time test predictions and errors: **(A)** electrical resistance and correlated strain of unseen test set vs. time; **(B)** unseen test set error distribution; **(C)** Measured strain vs. predicted strain for unseen test set.

Figure 8A shows some of the unseen test data that correlates measured resistance with measured strain. Using only unseen resistance data and the resistance gradient data as inputs for the trained LSTM resulted in predicted strains of the flexible sensor with an MAE of 19.29% strain. Figure 8B shows the comparison between predicted and measured. The error distribution can be seen in Figure 8C, which did show some errors up to 65%. The correlation of predicted vs. actual for the test set was 0.80, and increase of 0.23 compared to the correlation of raw resistance vs. actual, which was 0.67. This shows a significant increase in linearity of the system.

### Techniktex P130+B

**Figure 9:**
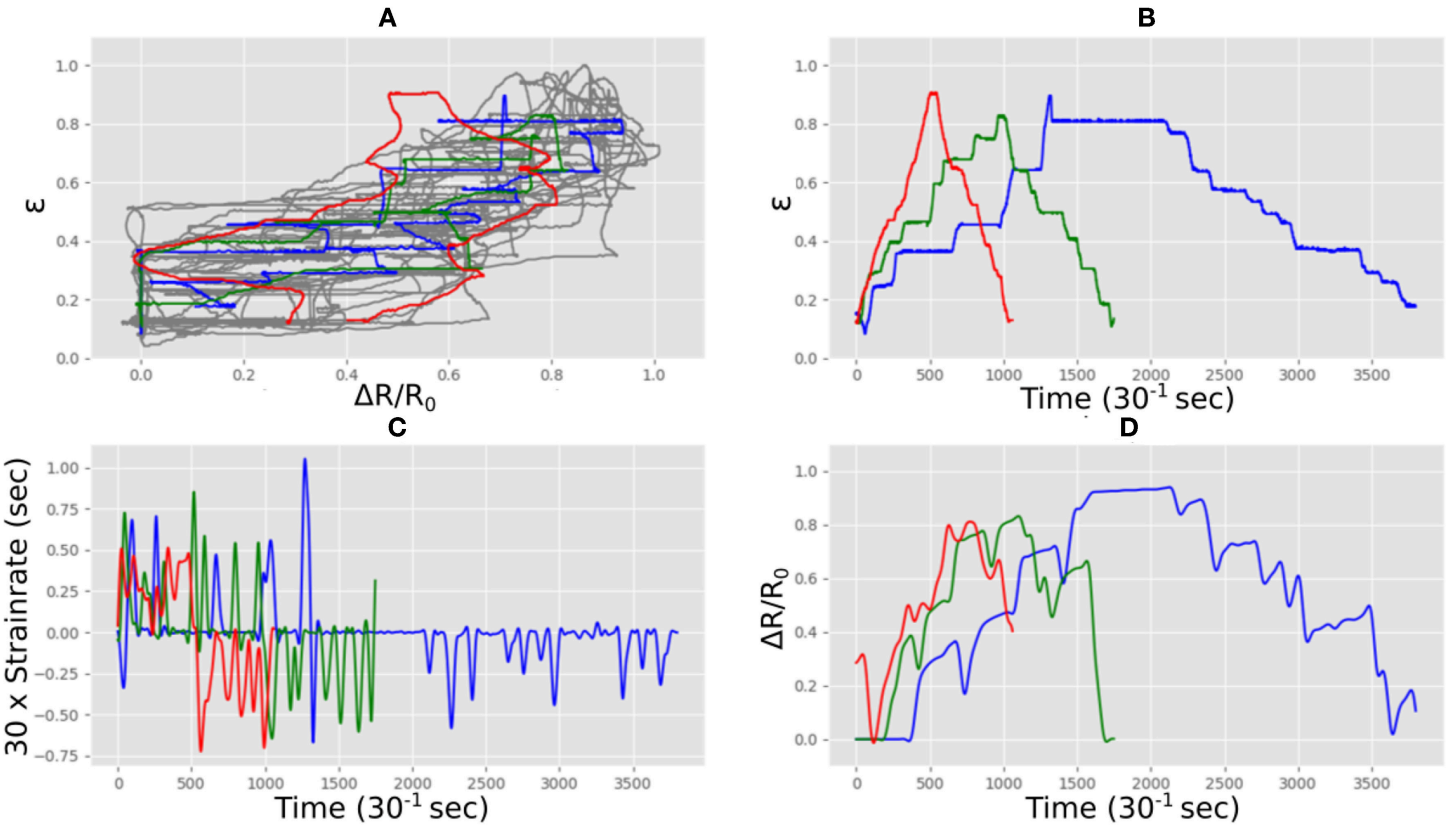
Strain and electrical resistance behavior of the Techniktex P130+B during stretching; **(A)** normalized strain vs. normalized resistance; **(B)** normalized strain vs. time; **(C)** strain rate vs. time; **(D)** normalized resistance vs. time.

The sensor was manually stretched and relaxed for ~15 min under the webcam, producing ~27,500 data points of correlations of measured sensor length vs. measured electrical resistance. As with the Medtex P130+B fabric, the complex nature of this data can be seen in can be seen in Figure 9A where three stretch and relax cycles for the sensor with three different rates (depicted in red, blue and green) produced very different functional forms. Figure 9B shows the output of the webcam correlated with the measured electrical resistance in each case showing that the rates were different in each case as quantified in Figure 9C. The rate of change of resistance for each case is shown in Figure 9D which was used as an input for the LSTM neural network.

**Figure 10:**
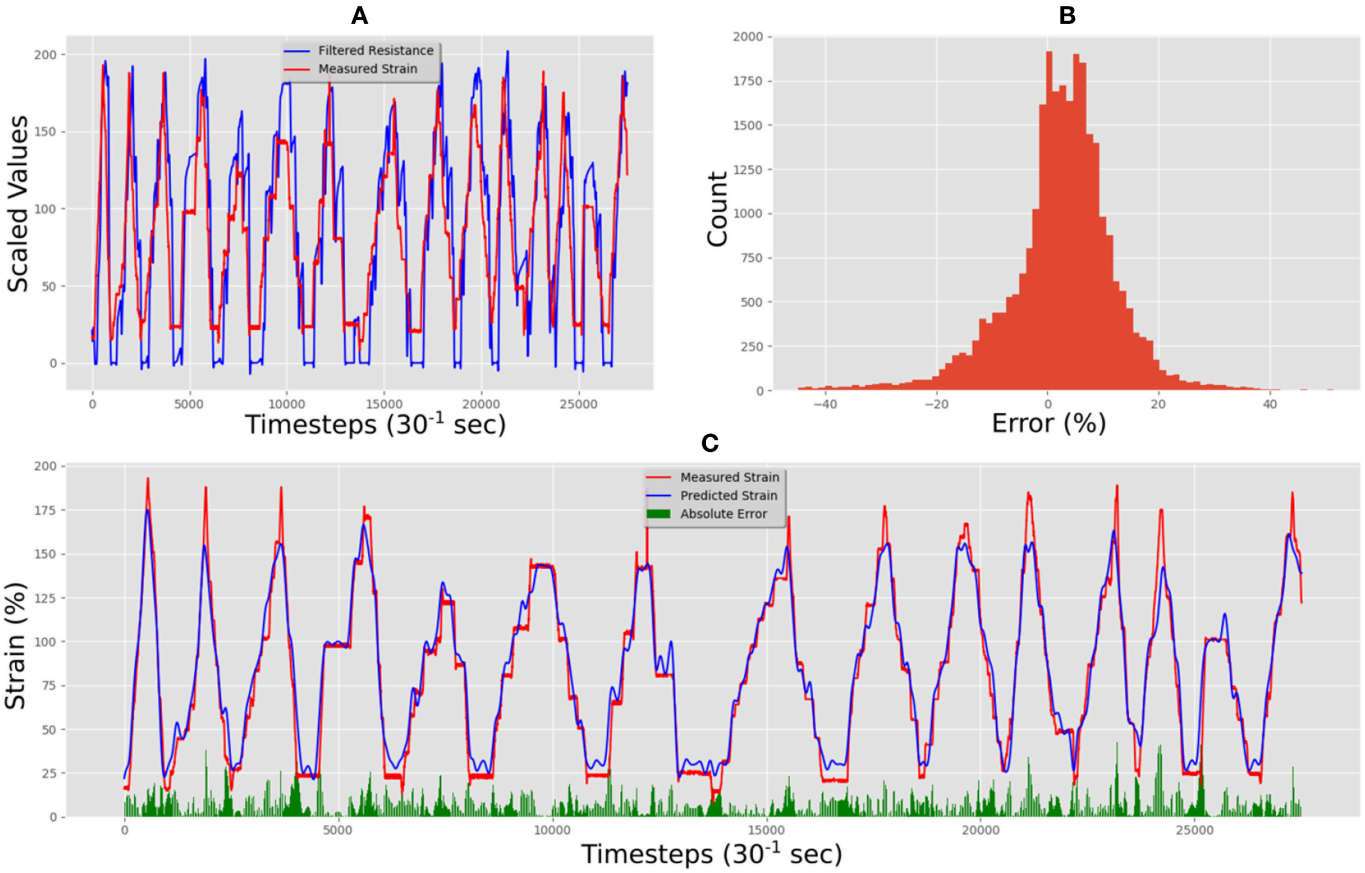
Techniktex P130+B training data predictions and errors: **(A)** electrical resistance and correlated strain of training set vs. time; **(B)** training set error distribution; **(C)** Measured strain vs. predicted strain for training set.

This dataset was used to train our LSTM architecture for around 17 h following an automated training schedule. The correlation between measured and resistance of the training data set can be seen in Figure 10A. The resulting strainpredictions can be seen in Figure 10B, where the Mean Absolute Error (MAE) was 6.64% total, and the error distribution can be seen in Figure 10C.

**Figure 11:**
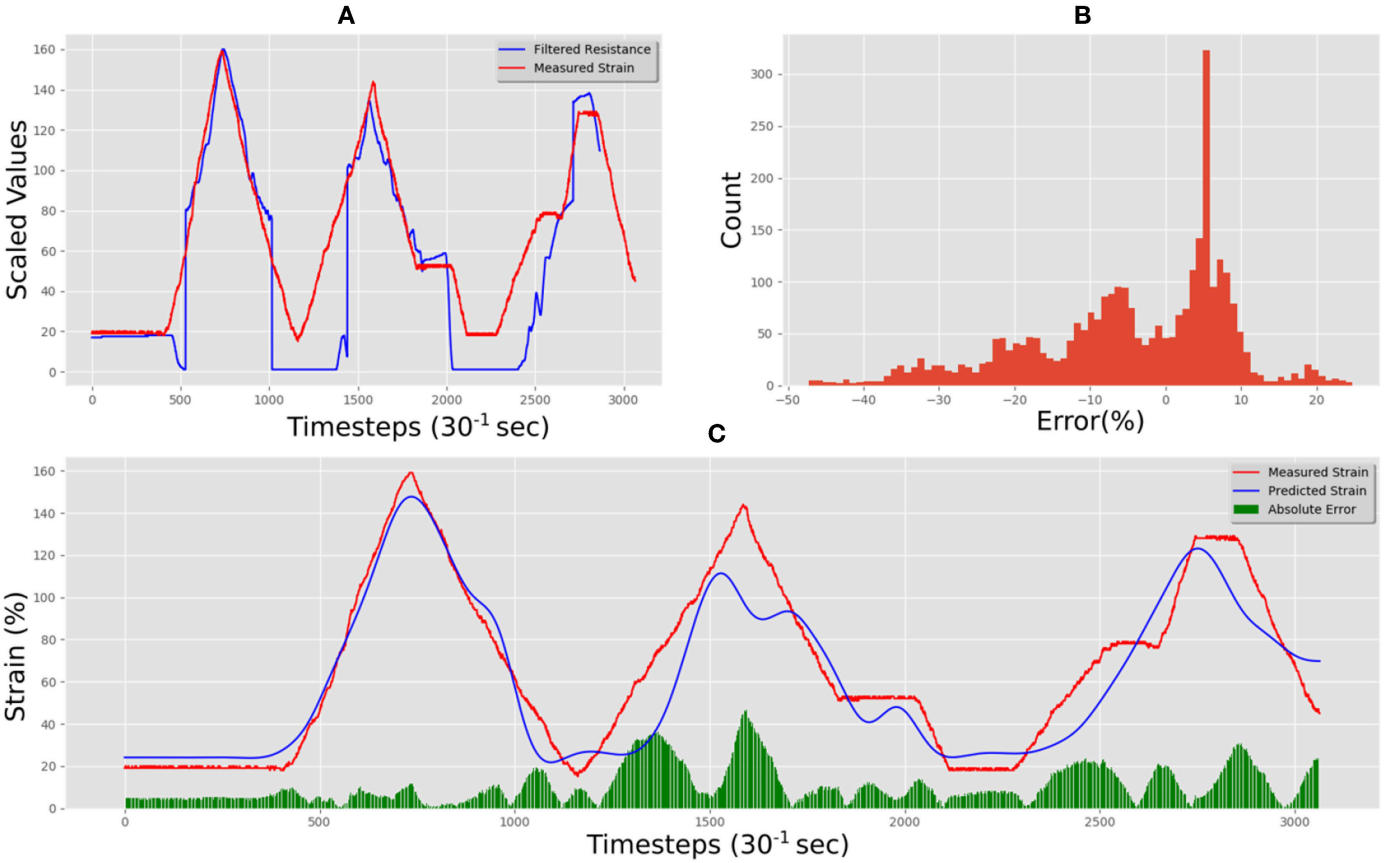
Techniktex P130+B real-time test predictions and errors: **(A)** electrical resistance and correlated strain of unseen test set vs. time; **(B)** unseen test set error distribution; **(C)** Measured strain vs. predicted strain for unseen test set.

Figure 11A shows the unseen test data that correlates measured resistance with measured. Using only the resistance data and the resistance gradient data as inputs for the trained LSTM resulted in predicted strain of the flexible sensor with an MAE of 10.75%. Figure 11B shows the comparison between predicted and measured. The error distribution can be seen in Figure 11C, which did show some errors up to 50%. The correlation of predicted vs. actual for the test set was 0.94, an increase of 0.09 when compared to the correlation of raw resistance vs. actual, which was 0.85.

### Adafruit Conductive Rubber

**Figure 12:**
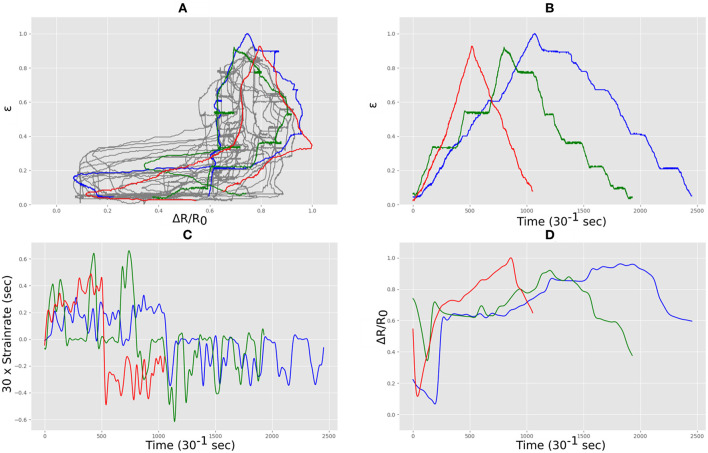
Strain and electrical resistance behavior of the Adafruit carbon-black rubber during stretching; **(A)** normalized strain vs. normalized resistance; **(B)** normalized strain vs. time; **(C)** strain rate vs. time; **(D)** normalized resistance vs. time.

The sensor was manually stretched and relaxed for ~15 min under the webcam, producing ~27,500 data points of vs. resistance. This material behaved in a more regular manner than the other two materials when exposed to different rates as shown in Figure 12A where three stretch and relax cycles for the sensor with three different rates are depicted in red, blue and green. Figure 12B shows the output of the webcam which measured the that correlated with the measured electrical resistance in each case showing that the rates as quantified in Figure 12C. The rate of change of resistance for each case is shown in Figure 12D which was used as an input for the LSTM neural network.

**Figure 13:**
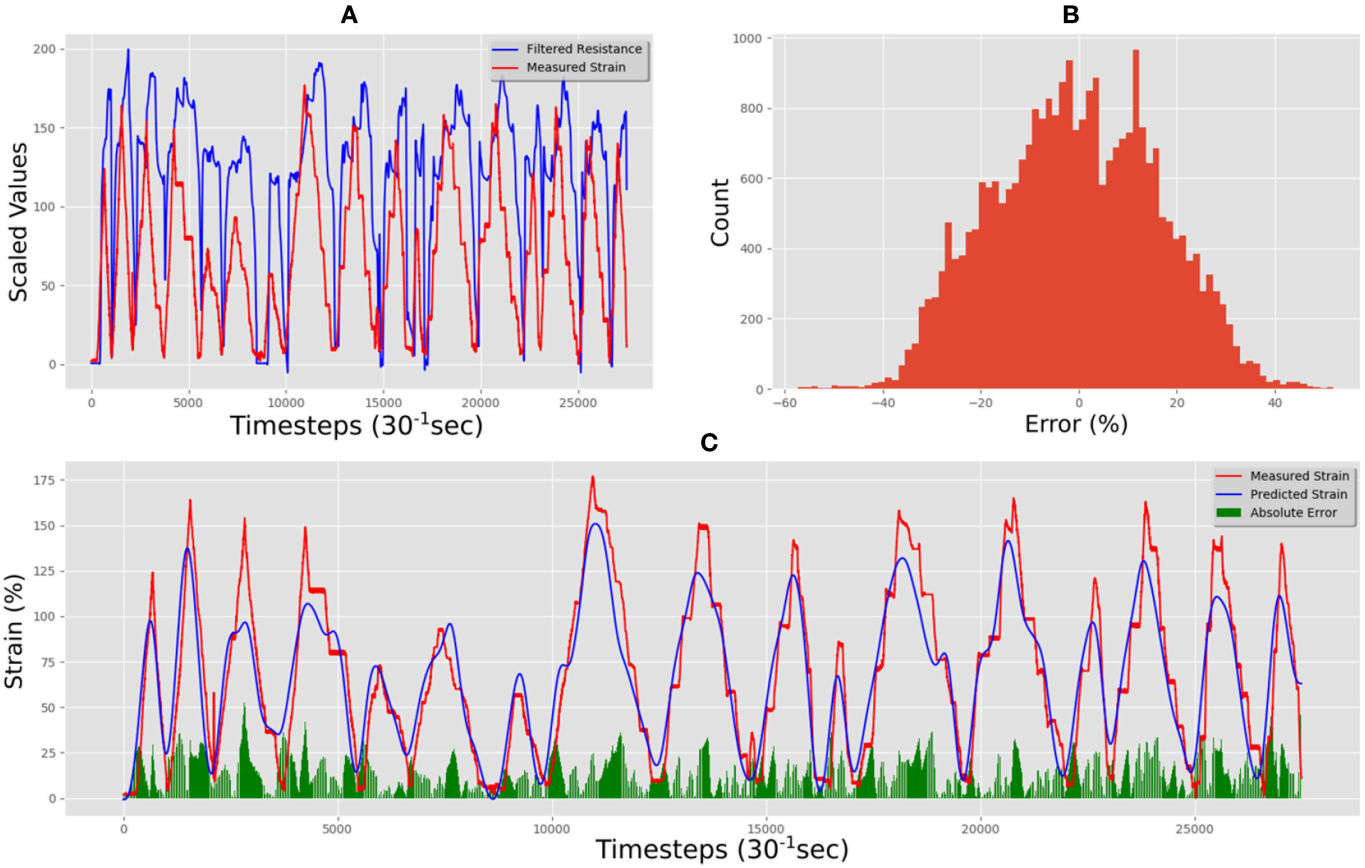
Adafruit carbon-black rubber training data predictions and errors: **(A)** electrical resistance and correlated strain of training set vs. time; **(B)** training set error distribution; **(C)** Measured strain vs. predicted strain for training set.

This dataset was used to train our LSTM architecture for around 13 h following an automated training schedule until the error did not improve further. The correlation between measured and resistance of the training data set can be seen in Figure 13A. The resulting predictions fitting the training data set can be seen in Figure 13B, where the Mean Absolute Error (MAE) was 13.77% total, and the error distribution can be seen in Figure 13C.

**Figure 14:**
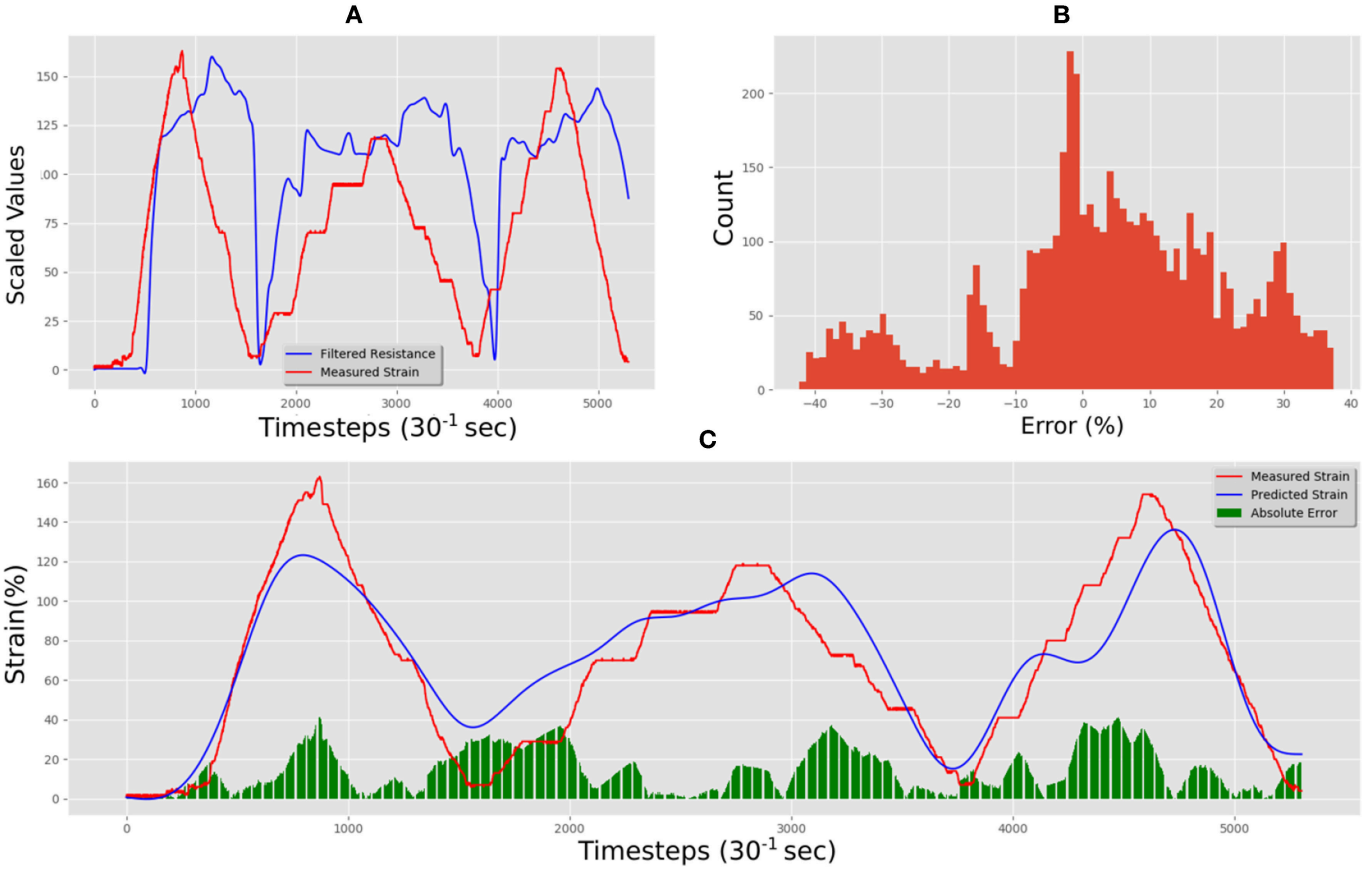
Adafruit carbon-black rubber real-time test predictions and errors: **(A)** electrical resistance and correlated strain of unseen test set vs. time; **(B)** unseen test set error distribution; **(C)** Measured strain vs. predicted strain for unseen test set.

Figure 14A shows the unseen test data that correlates measured resistance with measured strain. Using only the resistance data and the resistance gradient data as inputs for the trained LSTM resulted in predicted strain of the flexible sensor with an MAE for the test set was 14.49%. Figure 14B shows the comparison between predicted and measured. The error distribution can be seen in Figure 14C, which did show some errors up to 40%. The correlation of predicted vs. actual for the test set was 0.92, an increase of 0.32 compared to the correlation of raw resistance vs. actual, as the correlation was only 0.60 for this material-a highly significant increase.

### Comparison With Other Statistical Methods

To evaluate the effectiveness of our deep learning approach we tested four alternative methods for calculating strain from the sensor data. These were: a standard linear regression model (Schneider et al., 2010); a 5th degree polynomial regression model (Heiberger and Neuwirth, 2009); a deep feed-forward neural network (DNN) (Schmidhuber, 2015); and a traditional recurrent neural network (RNN) (Jain and Medsker, 2000). Both the network models have the same structure as our LSTM network, that of 3 layers of 270 nodes. These were fitted to the training data from all three materials and tested on the unseen datasets, in the same manner as our LSTM method. The regression models were created using the SciPy package (Version 0.19.1), and the networks were created using the Keras package (Version 2.2.2).

We can see from Table 1 that the LSTM out-performs the simpler methods consistently for all three sensor types. The LSTM results show an average improvement of 10.0% error compared to the linear models, and an average improvement of 3.4% error on unseen test data compared to the simpler RNN. The linear and polynomial regression models perform poorly which is not surprising given the complexity of the non-linearity of the sensors. Although potentially capable of modeling much greater complexity, the DNN performs worse than the linear and polynomial regression methods, this is likely to be due to its single input/single output structure. As soon as some recurrence is added to the network architecture, as with the RNN and the LSTM, the results are greatly improved with a significant drop in error across all three materials, however generally a greater difference in error between the training data and the testing data is seen. This suggests that some overfitting is present, and that significant improvements can still be made in the future.

**Table 1:** Table of results comparing different methods for calculating strain from the measured resistance value.

	**Medtex P130+B**	**Techniktex P130+B+B**	**Adafruit rubber**
**Linear regression model**			
*Training Set Prediction Error (MAE%)*	19.3	17.5	28.6
*Test Set Prediction Error (MAE%)*	24.8	19.3	30.6
**5th deg polynomial regression model**			
*Training Set Prediction Error (MAE%)*	18.9	17.2	27.0
*Test Set Prediction Error (MAE%)*	25.0	19.2	28.9
**Deep feedforward neural network**			
*Training Set Prediction Error (MAE%)*	23.2	20.1	28.3
*Test Set Prediction Error (MAE%)*	25.1	21.3	32.1
**Recurrent neural network**			
*Training Set Prediction Error (MAE%)*	16.4	11.8	17.1
*Test Set Prediction Error (MAE%)*	22.1	14.2	18.3
**LSTM neural network**			
*Training Set Prediction Error (MAE%)*	11.4	6.64	13.8
*Test Set Prediction Error (MAE%)*	19.3	10.8	14.5

## Discussion

In general, wearable technology developers would prefer perfectly linear flexible sensors, however such sensors have historically been difficult to make hence our approach to use available commercial flexible sensors and use deep learning algorithms such as LSTMs to make them usable in wearable technology. To test our approach we used three commercially available flexible sensors all of which showed non-linearity and strain rate dependant electrical responses. The summary of our results in Table 2 shows that we can predict strain to between 10 and 20% error for three different sensor types. The origin of non-linearity and rate dependence in flexible sensors is different in each case, and hence they show very different behavior. However, there are some general principles which our selection of commercial sensors illustrate.

**Table 2:** Summary of results of the three commercially available stretch sensors used in this study.

	**Medtex P130+B**	**Techniktex P130+B+B**	**Adafruit rubber**
No. of training examples	27500	27500	27500
No. of testing examples	5000	3200	5300
Raw training set correlation	0.67	0.85	0.60
Training set prediction correlation	0.95	0.98	0.93
Test set prediction correlation	0.80	0.94	0.92
Training set prediction error (MAE%)	11.38	6.64	13.77
Test set prediction error (MAE%)	19.29	10.75	14.49

The fabric sensors have a macrostructure comprising of a warp and weft. In these sensors the conductive route through the material is via many different temporary mechanical connections which arise where the conductive fibers in the warp touch the weft and in doing so make another potential conductive path through the material. During stretch these local connections change both in number and area of contact, and this changes the electrical resistance. The geometry of the fabric macrostructure during different stretch does not scale linearly with extension and so it is not surprising that fabric sensors are non-linear in their electrical response. Similarly on release their electrical properties are dependent on the way the individual fibers unstretch and mechanically slide past each other. Although the topology of the warp and weft remains in tact after stretch, at the microscale of the individual connective fibers, different mechanical connections and adjacencies result once the fabric returns to its original length. Hence the return path of de-stressing such a fabric is likely to be different. In addition the elastomer component of the fibers are viscoelastic and so their mechanical response is highly sensitive to strain rate. The difference between the two fabrics arises from thier intended use. Techniktex is designed with sensor applications in mind, having homogeneous conductivity in all directions, and more reliable and linear behavior. The Medtex is primarily aimed at medical dressings, meaning that the homogeneity of the conductivity is not a major manufacturing concern. All these factors taken together produce the highly non-linear and distinctly different behaviors seen in our fabric sensors shown in Figures 6A, 9A.

The Adafruit conductive rubber sensor is comprised of a viscoelastic rubber material with carbon black powder added. Here the electrical conduction arises from a percolation path of carbon particles in contact with each other. During stretching the percolative path changes as particle contact changes. As with the conductive fabrics the matrix elastomer is viscoelastic and so the combination of microscale contacts changing with length with rate dependant restoration forces results in a high non-linear electrical response. An interesting result arises with the conductive rubber as although the raw data has a much lower correlation than the two fabrics, it achieves an impressive increase after training, although still with a larger overall error than the Techniktex fabric. We speculate that this is due to the lack of macroscale structure (warp and weft) in the rubber which allows the behavior to be learnt much more affectively by the neural network. This has some interesting implications for the inevitable use of deep learning with soft materials. Previous to the very recent increase in use of learning algorithms, structure and complexity has been focused on the physical geometries and structure, balanced against the human-limited ability to efficiently produce applicable models. Now that the limits of applied models has changed, with a huge increase in the complexity of non-human designed models of correlating input to output without the need for the intermediary steps to be established in full, the way we approach our use of materials may change. If the most powerful element in a system is its own learning capability, then physical design may begin to change in accordance. We find here that comparable results are possible with the simplest of physical approaches compared to a highly refined composite textile structure.

The training time of the LSTM on our system, which comprised of a simple low-spec PC, was of the order of 10–20 h, but this could be reduced significantly through software optimisation and the use of faster machines. Nevertheless, this amounts to a calibration process which only needs to be performed once. The more stringent criterion for real use application requires the trained LSTM to give real time high resolution values of stretch, and this requires a portable resistance measurement circuit and a portable computation unit to run the LSTM, as illustrated in Figure 1. We estimate the requirement for these would be entirely feasible.

There are many advantages of a deep learning approach to calibration that have not been applied here. A major avenue which we will investigate in the future is “transfer learning,” where pre-trained models are applied to new datasets, either for direct inference, or for reductions in training time. For our application, this would be expected to be useful for different geometries of the same or similar materials. Another advantage of using this approach concerns wear and damage of sensors. With additional new datasets, an original trained system might be able to adjust itself to damage, thereby prolonging the usefulness of sensors that generally require considerable manual construction. For instance (Graves et al., 2005) have shown LSTM networks are able to re-train rapidly to adapt to new subsets of data achieving greater accuracy than when trained from scratch.

## Conclusion

We have developed a deep learning method for calibrating highly hysteretic resistive stretch sensors. We show that technique gives reliable robust strain information for commercially available textile and elastomeric stretch sensors and requires no specialist equipment. Our LSTM model is more accurate than four other statistical models tested, as shown by consistent significantly lower errors on unseen datasets. Our method is open source and does not require any a priori knowledge of the physical attributes or geometry of the sensor to be calibrated, which is a key advantage as stretchable sensors are generally applicable to highly complex geometries with integrated electronics requiring bespoke manufacture.

## Author Contributions

BO, PS, and MM conceived the project. BO carried out the work, building and testing the prototype sensors and the deep learning software. RJ helped with the building and testing the prototype sensors. MM and BO analyzed the data and wrote the paper.

### Conflict of Interest Statement

The authors declare that the research was conducted in the absence of any commercial or financial relationships that could be construed as a potential conflict of interest.
